# LSTM4piRNA: Efficient piRNA Detection in Large-Scale Genome Databases Using a Deep Learning-Based LSTM Network

**DOI:** 10.3390/ijms242115681

**Published:** 2023-10-27

**Authors:** Chun-Chi Chen, Yi-Ming Chan, Hyundoo Jeong

**Affiliations:** 1 Department of Electrical Engineering, National Chiayi University, Chiayi 600, Taiwan; 2 MindtronicAI Co., Ltd., Taipei 116, Taiwan; yimingchan@gmail.com; 3 Department of Mechatronics Engineering, Incheon National University, Incheon 22012, Republic of Korea

**Keywords:** Piwi-interacting RNA (piRNA), RNA prediction, machine learning, LSTM

## Abstract

Piwi-interacting RNAs (piRNAs) are a new class of small, non-coding RNAs, crucial in the regulation of gene expression. Recent research has revealed links between piRNAs, viral defense mechanisms, and certain human cancers. Due to their clinical potential, there is a great interest in identifying piRNAs from large genome databases through efficient computational methods. However, piRNAs lack conserved structure and sequence homology across species, which makes piRNA detection challenging. Current detection algorithms heavily rely on manually crafted features, which may overlook or improperly use certain features. Furthermore, there is a lack of suitable computational tools for analyzing large-scale databases and accurately identifying piRNAs. To address these issues, we propose LSTM4piRNA, a highly efficient deep learning-based method for predicting piRNAs in large-scale genome databases. LSTM4piRNA utilizes a compact LSTM network that can effectively analyze RNA sequences from extensive datasets to detect piRNAs. It can automatically learn the dependencies among RNA sequences, and regularization is further integrated to reduce the generalization error. Comprehensive performance evaluations based on piRNAs from the piRBase database demonstrate that LSTM4piRNA outperforms current advanced methods and is well-suited for analysis with large-scale databases.

## 1. Introduction

The Piwi-interacting RNAs (piRNAs) are a new class of small, endogenous, non-coding RNAs (ncRNAs) that regulate gene expression through various mechanisms. These piRNAs can further interact with Piwi-class proteins to form the piRNA-induced complexes that silence transposons in germline cells. Research has demonstrated that piRNAs are associated with the control of transposon silencing, epigenetic regulation, and RNA silencing in diverse organisms [1,2,3]. Furthermore, recent studies have linked piRNAs to virus defense, as well as to the development and progression of many types of cancer [4,5,6,7]. Due to their potential as therapeutic targets for certain diseases, there is a growing interest in identifying novel piRNAs. Therefore, efficient computational methods are required for large-scale piRNA detection to accelerate piRNA exploration.

PiRNAs are the largest class of small ncRNAs, typically ranging in sequence length from 24 to 33 nucleotide bases [8,9]. However, piRNAs lack conserved structure motifs and sequence homology across species, which makes it challenging to recognize them [10]. Currently, there are two main classes of piRNA detection methods. The first class utilizes sequence-based features from known piRNAs to predict new ones. While piRNAs tend to have a uridine base at the first position and an adenosine base at the tenth position, relying solely on the base position to predict piRNAs is not accurate [11]. The k-mer scheme approach, piRNAPredictor [12], takes the frequencies of k-mer features and linear discriminant analysis (LDA) to predict piRNAs with better performance. Furthermore, piRNAdetect [13] enhances the prediction accuracy by using N-gram models along with a support vector machine (SVM) to parse and classify the piRNAs. By using the deep learning technique, piRNN [14] first summarizes the k-mer motifs based on their base positions and creates a corresponding feature vector with 1364 items. The feature vector is then normalized and fed into a convolutional neural network (CNN) to make sound predictions for piRNAs. In addition, GAWE [15] utilizes ensemble weight learning for the combined features and random forest classification to predict piRNAs with high accuracy. As some piRNAs have been found to be derived from genomic piRNA clusters, the second class of piRNA detection methods employs the genomic features of the clustering locus to predict piRNAs. Several clustering-based methods have been developed to predict clustered piRNAs [16,17], and the clustering features can further be incorporated with sequence-derived features by using multiple kernel SVM [18,19]. However, it should be noted that clustering-based approaches only work for clustered piRNAs and that some databases may not provide clustering information.

As the amount of genomic data continues to surge, analyzing extensive databases has become increasingly challenging. A prime example is the piRBase database, which has seen staggering growth in the number of sequences of human piRNAs from 32,000 [9] to over 8.5 million [20]. However, there is a lack of effective tools capable of analyzing such a large database with millions of data for piRNA prediction. To bridge this gap, we have developed a sequence-based detection algorithm, called LSTM4piRNA, that employs long short-term memory (LSTM) to predict piRNAs accurately. Unlike previous approaches that manually select features from known piRNAs, LSTM4piRNA can automatically extract and learn useful features from a large database to maximize detection performance. The performance results based on the piRNAs in the piRBase [9,20] database show that LSTM4piRNA outperforms previous algorithms in terms of efficiency and accuracy. Furthermore, we have developed a web server that allows users to easily predict piRNAs through LSTM4piRNA. The user can submit RNA sequences in the FASTA format to the server and check the predicted results.

## 2. Results

To evaluate the performance of the proposed LSTM4piRNA, we conducted four-fold cross-validation experiments using constructed datasets on piRNAs from four species: *H. sapiens*, *R. norvegicus*, *M. musculus*, and *C. elegans*. The dataset was evenly divided into four subsets. Each subset was used as the testing set in turn, while the remaining subsets were used for training. The performance of piRNA detection is assessed in terms of the accuracy (ACC) = (TP+TN)(TP+TN+FP+FN), the sensitivity (SEN) = $\frac{\mathrm{TP}}{\mathrm{TP}+\mathrm{FN}}$, and the positive predictive value (PPV) = $\frac{\mathrm{TP}}{\mathrm{TP}+\mathrm{FP}}$. TP denotes the number of piRNAs correctly identified, while TN denotes the number of correctly identified negative samples. FP represents the number of negative samples mistakenly classified as piRNAs, and FN represents the number of piRNAs missed in the detection process. In addition, the harmonic metric F-score = $2/(\frac{1}{SEN}+\frac{1}{PPV})$ is also employed to assess the performance.

### 2.1. Regularization and Generalization

**Figure 1:**
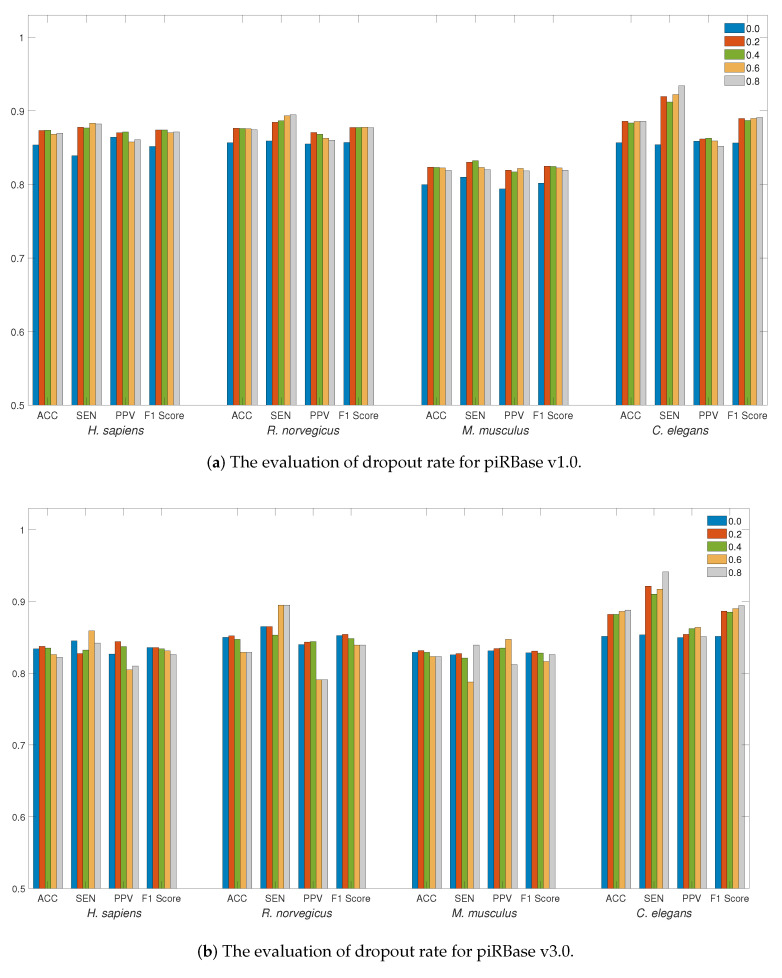
The evaluation of detection performance on piRBase datasets with regard to regularization. Bars in different colors correspond to the test for different species. The ACC, SEN, PPV, and F1-Score are illustrated for comparison.

We first evaluated the influence of the dropout rate on the piRNA dataset. The use of dropout regularization in neural networks serves as a preventive measure against overfitting. The detection performance at different dropout rates, ranging from 0 to 0.8 with a step size of 0.2, is illustrated in Figure 1a,b. The adoption of dropout regularization can enhance the detection performance across a range of dropout rates for the piRBase v1.0 dataset. For the piRBase v3.0 dataset, a dropout rate between 0.2 and 0.4 proves to be most effective, especially when accuracy is the main consideration, except for the *C. elegans* species. Given that the *C. elegans* dataset is relatively smaller, a higher dropout rate can mitigate overfitting on training data and enhance detection performance. Selecting an appropriate dropout rate is essential for reducing generalization error. Therefore, for piRNA detection in LSTM4piRNA, we have set the dropout rate to 0.2.

**Figure 2:**
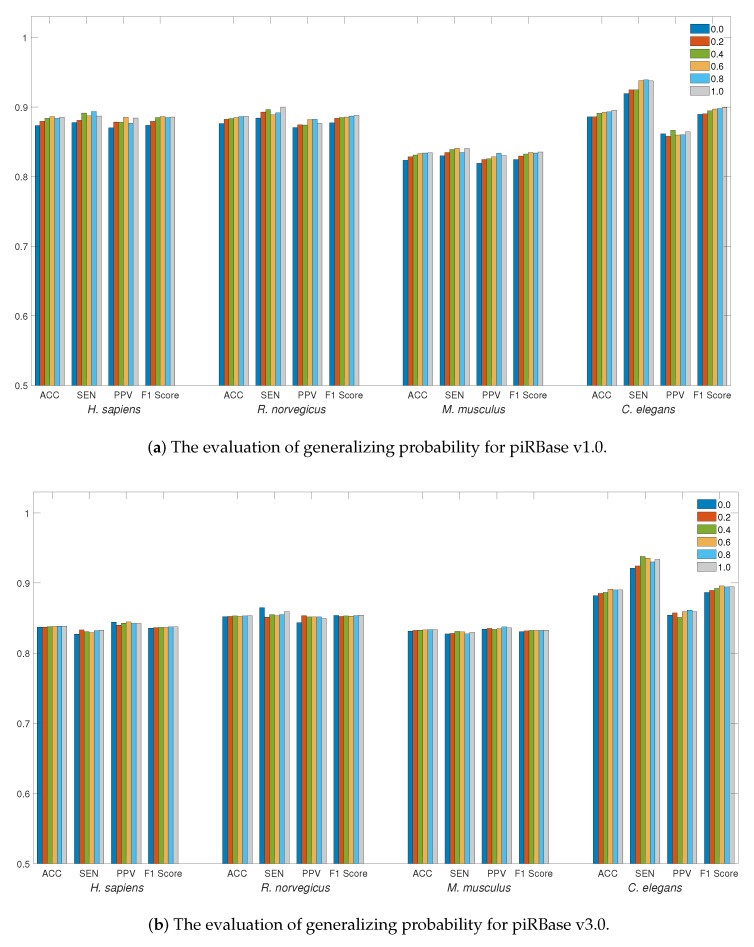
The evaluation of detection performance on piRBase datasets with regard to generalization. Bars in different colors correspond to the test for different species. The ACC, SEN, PPV, and F1-Score are illustrated for comparison.

For data generalization, Figure 2a,b illustrate the accuracy of data generalization at various probabilities ranging from 0 to 1.0 with a step size of 0.2. Employing data generalization can effectively reduce generalization errors, leading to a more robust training model. This strategy can result in more substantial improvements to the piRBase v1.0 dataset, particularly when working with limited data quantities. For all species, generalizing probabilities greater than 0.4 can offer better accuracy, with particular efficacy observed for the *C. elegans* dataset. To augment piRNA detection, LSTM4piRNA has set the generalizing probability to 0.6.

### 2.2. Accurate Prediction of piRNA Sequences

We evaluated the effectiveness of LSTM4piRNA against the following algorithms: piRNAPredictor, GAWE, and piRNN. For the performance comparison, piRNAPredictor, GAWE, and piRNN with default settings were evaluated on the same test datasets. The evaluation results and total run time (in seconds) for each species are summarized in Table 1 and Table 2. The computation time was measured on a 64-bit server machine running Linux kernel 5.8.0 with 8-core CPUs clocked at 3.5 GHz and 32 GB RAM.

Except in terms of the sensitivity metric for *R. norvegicus* species, LSTM4piRNA consistently outperforms the other methods in most evaluation metrics for the piRBase v1.0 dataset. It achieves the highest accuracy, PPV, and F-score (which evaluates the harmonic mean of SEN and PPV), as illustrated in Table 1.

While GAWE exhibits higher sensitivity for the *R. norvegicus* species, it comes with significantly higher time complexity due to its adoption of an ensemble and iterative framework. In comparison, LSTM4piRNA’s sensitivity for *R. norvegicus* is nearly on par with GAWE, with only a minor performance discrepancy. Moreover, although LSTM4piRNA achieves the highest performance scores across most test cases, it demands the least computation time for predictions. On the other hand, while piRNN exhibits promising prediction performance, its computation time is nearly tenfold compared to our proposed algorithm, as piRNN has a larger number of neurons and filters, making it less suitable for analyzing large-scale datasets. Although deep learning-based approaches exhibit improved performance metrics for piRNA detection, they may entail significant computational costs. Therefore, careful design of effective deep neural networks is essential to achieve promising performance while maintaining reasonable computational expenses.

Based on the performance assessment using the piRBase v3.0 datasets, we confirm that LSTM4piRNA achieves higher performance metrics across all species, with a remarkable performance gap as shown in Table 2. Please note that, in our performance evaluation on piRBase v3.0, we only obtained the analysis results for LSTM4piRNA and piRNAPredictor due to the limited computational resources. However, we are able to assess all algorithms for *C. elegans* because its dataset size is relatively smaller compared to that of other species. Based on the simulation results, we can verify that LSTM4piRNA has better scalability than piRNN, which adopts convolutional neural networks to predict piRNAs. Additionally, we confirm that LSTM4piRNA requires less computation time compared to other algorithms, while it achieves distinctly higher ACC, SEN, PPF, and F1-scores.

Overall, LSTM4piRNA can automatically learn the critical features and provide superior prediction performance compared to approaches that use artificial feature selection. Furthermore, LSTM4piRNA has the fastest prediction efficiency among all compared algorithms for all datasets.

## 3. Discussion

Due to the lack of distinct characteristics for piRNA identification, accurate prediction of piRNAs poses a significant challenge. Most existing piRNA detection methods predominantly rely on machine learning techniques that necessitate manual feature selection. However, this approach may overlook critical features or incorporate irrelevant data, leading to suboptimal prediction performance. To address this issue, we introduce LSTM4piRNA, a novel computational approach for piRNA detection, and also develop a web-based application for piRNA analysis. By leveraging LSTM networks, LSTM4piRNA can autonomously learn sequence characteristics from unstructured data and incorporate generalization and regularization to enhance model resilience. Through extensive performance assessments using piRNAs from the piRBase database, LSTM4piRNA has demonstrated impressive accuracy, outperforming all other existing algorithms in piRNA detection. Moreover, LSTM4piRNA is a time-efficient algorithm that can efficiently process large-scale databases, and its use can be further extended to analyze other similar databases. To make the software more accessible, we offer a web server version of LSTM4piRNA, ensuring it’s available to researchers who may not have a strong software background or ample computational resources. The algorithm we propose carries significant potential to advance the field of piRNA research and aid in exploring their clinical applications.

The performance of piRNA detection is generally influenced by both the characteristics of the training dataset and the prediction model. To achieve good performance in a sequence-based approach, it is essential to have a large enough training dataset to cover all species and a prediction model capable of learning the representative features of the dataset. Although most machine learning approaches use artificial feature selection techniques to extract features from the dataset, this may not be suitable for large datasets due to the difficulty in identifying all significant features. In contrast, LSTM4piRNA can efficiently handle large datasets and automatically learn the critical features. This method can also be extended to analyze other databases with vast amounts of sequential data for further analysis. As evidenced in this study, LSTM4piRNA successfully leverages LSTM for feature extraction, leading to effective prediction of piRNAs and exhibiting exceptional prediction performance on both the piRBase v1.0 and v3.0 datasets.

While genome sequences are a fundamental feature commonly used in genomic analysis, LSTM4piRNA cannot fully predict all piRNAs due to biological diversity. To overcome the limitation, integrating biological priors such as genomic loci and regulatory network models into the algorithm would be an appropriate direction for future studies. Note that not all genomic sequences come with such biological prior information, and the integration might also require the adoption of additional machine learning techniques. Furthermore, incorporating different artificial intelligence frameworks into the piRNA prediction algorithm would facilitate a deeper understanding of the roles of specific piRNA sequences.

## 4. Materials and Methods

Effective identification of piRNAs through genome sequences requires a mechanism that can learn the relevant features within the sequences. However, manually summarizing and consolidating general features from piRNAs, as attempted in previous studies, may lead to overfitting or the exclusion of important features. As the LSTM network is designed to model chronological sequences, it can automatically learn both long-term and short-term dependencies over the sequences [21,22]. Thus, LSTM networks are applicable to a variety of sequential problems, including speech processing, grammar learning, and semantic parsing. For the sake of piRNA detection, each nucleotide base is encoded into a one-hot vector, and these vectors are then concatenated into a vector sequence. We apply the LSTM network to this concatenated vector sequence to uncover correlations across the sequences, thus transforming the input sequence into informative base embedding. Based on the embedded representation of the input sequence, we employ a feedforward neuron network to determine the class of the input sequence. Moreover, regularization and generalization methods are employed to minimize generalization errors. The detailed procedure for piRNA detection using the LSTM4piRNA method is presented in the following subsections.

### 4.1. Encode Data and Generalization

To train and evaluate piRNA detection, we download piRNAs from the piRBase v1.0 [9] and v3.0 databases [20] for species including *Homo sapiens*, *Caenorhabditis elegans*, *Rattus norvegicus*, and *Mus musculus*. The piRNAs with lengths ranging from 18 to 40 are randomly drawn from piRBase as positive samples. Note that, in the piRBase database, the majority of piRNAs fit within the 18–40 nucleotide range, with only a few exceptions outside this range, and the proportion is exceptionally low. The maximum sample size is set to 100,000 for the piRbase v1.0 dataset and 1,000,000 for the expanded piRbase v3.0 dataset. Table 3 summarizes the total number and average length of the piRNAs for each species in the constructed benchmark. To create the negative samples, ncRNAs are first taken from the Rfam 14.6 database [23,24]. For each sequence in the positive samples, the sub-sequence with the same length is randomly drawn from the Rfam database and shuffled to create the negative control sample. Based on the aforementioned strategy for generating the benchmarking dataset, each positive sample has a matching negative control sample so that we have the exact same number of positive and negative samples. Next, we encode each base of the sequence into a 4-bit one-hot vector according to Table 4. For example, the RNA sequence {ACCG} is encoded into the vectors {[1,0,0,0], [0,0,1,0], [0,0,1,0], [0,0,0,1]}. Following the one-hot encoding, since 4 bits are required to represent a single nucleotide base, the input sequence of length *L* is converted to a sequence of vectors with a total size of L×4. These one-hot vectors are then sequentially fed into the LSTM network. During the training phase, we introduce data generalization by randomly reordering the negative samples with a generalizing probability. This data generalization allows the model to adapt effectively to new data, thereby enabling the LSTM network to learn more relevant features and reduce potential generalization errors.

### 4.2. Network Architecture and Regularization

**Figure 3:**
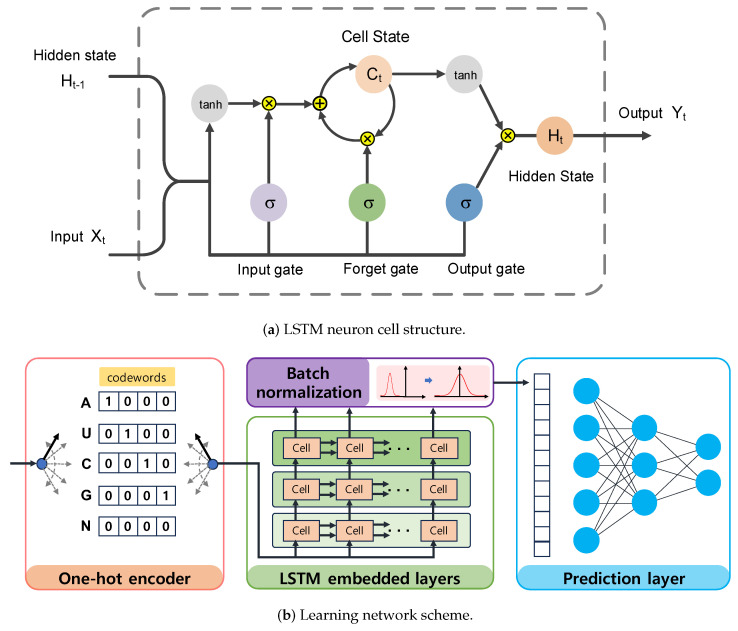
The LSTM4piRNA architecture. (**a**) The LSTM neuron consists of cell state $C_{t}$ and hidden state $H_{t}$, which are controlled by three gates. The σ gate is implemented using the logistic sigmoid function, while the tanh activation is the hyperbolic tangent function. Both addition and multiplication operations are performed point–wise. (**b**) The learning network schematic. The learning network includes regularization, three–layer LSTM neuron networks, batch normalization, and the feedforward neuron network.

The LSTM is an advanced type of recurrent neural network that can learn the dependencies of an entire input sequence by sharing weights and updating control states over time. As illustrated in Figure 3a, the LSTM neuron cell is mainly composed of the cell state and hidden state, which are controlled by three gates using the sigmoid function to memorize important information or discard less relevant information from prior data. Initially, the input gate of the LSTM network takes two input signals, including the previous hidden state $H_{t-1}$ and the current input $X_{t}$. It then determines which information should be updated to the cell state $C_{t}$ based on the output of the sigmoid function $\sigma \left(\cdot \right)$ and hyperbolic tangent function $\tanh\left(\cdot \right)$. The update rule for the input gate is given by

(1a) $$T_{a}\left(H_{t-1},X_{t}\right)=W\left.\cdot \left.[H_{t-1},X_{t}\right.]+B\right.$$

(1b) $$i_{t}=\sigma \left(T_{a}\left(H_{t-1},X_{t}\right)\right)$$

(1c) $$\tilde{C}=\tanh\left(T_{a}\left(H_{t-1},X_{t}\right)\right),$$

where $T_{a}$ represents an affine transform function with trainable weight *W* and bias *B* parameters. The operator $\left[\cdot \right]$ denotes vector concatenation. Furthermore, $i_{t}$ is the output of the sigmoid function, while $\tilde{C}$ is the output of the hyperbolic tangent function.

Subsequently, the forget gate decides what information should be discarded from the cell state, and the output of the forget gate is given by:

(2) $$f_{t}=\sigma \left(T_{a}\left(H_{t-1},X_{t}\right)\right).$$

As a result, the cell state $C_{t}$ for long-term memory can selectively retain part of the activated data using both the input gate and the forget gate. Specifically, the LSTM updates the cell state by combining the outputs of the input gate and forget gate as shown in the following equation:

(3) $$C_{t}=f_{t}*C_{t-1}+i_{t}*\tilde{C_{t}}.$$

The hidden state $H_{t}$ for short-term memory can then access the activated cell data through the output gate. The output gate selectively passes the information to the next cell based on the following equation:

(4) $$H_{t}=\sigma \left(T_{a}\left(H_{t-1},X_{t}\right)\right)*\tanh\left(C_{t}\right).$$

The data stored in the hidden state are taken as the neuron output and are also fed back to the input data across time steps.

LSTM4piRNA is primarily based on the LSTM algorithm, and its learning network architecture is illustrated in Figure 3b. Considering that piRNAs are relatively short sequences, the inclusion of deeper layers and hidden states does not significantly enhance performance but increases the risk of overfitting. To avoid the overfitting that often accompanies increased model complexity and ensure reliable generalization performance, we adopt a 3-layer LSTM with 32 hidden states. The streamlined model not only reduces computational demands but also enhances the ability to process large-scale data effectively. The initial step in LSTM4piRNA involves encoding the input sequence into one-hot vectors and generalizing the training sequences. Following this, the three-layer LSTM network processes the one-hot vectors sequentially to automatically learn and identify the features. The output from the last hidden state is subjected to batch normalization to reduce the covariate shift and provide additional regularization [25]. The encoded informative embedding is then directed into a single-layer feedforward neuron network of size 32×2 for piRNA prediction. In the final layer, the feedforward neural network outputs a 2-bit one-hot vector corresponding to the piRNA and non-piRNA predictions. During the training phase, we set the batch size to 128 and allow for a maximum of 300 epochs. We employ the Adam optimizer with a learning rate of 0.001 and use the cross-entropy loss function [26]. To circumvent overfitting during training, we apply dropout regularization to both the LSTM and feedforward networks, randomly disabling node connections with a specified probability. The dropout regularization enables the neurons to identify more robust features independently, thus avoiding over-reliance on other nodes and overfitting of the training data [27,28]. We empirically optimize the dropout rate and generalization probability, as depicted in Figure 1 and Figure 2. Additionally, the hyperparameters are set empirically based on our experiments, with the optimized values recommended for general use. However, users can freely adjust these hyperparameters to align with their preferences and the statistical properties of their training datasets.

## Figures and Tables

**Table 1:** Performance evaluation on the piRBase v1.0 dataset. Note that we highlight the best performer using boldface font.

Method	*H. sapiens*				
	ACC	SEN	PPV	F1-Score (%)	Log${}_{10}$ (Time)
LSTM4piRNA	**88.66**	**89.86**	**87.75**	**88.79**	**1.05**
piRNAPredictor	77.79	81.36	75.94	78.56	1.76
GAWE	80.35	82.13	79.31	80.70	3.66
piRNN	86.88	87.82	86.20	87.00	2.24
	***R. norvegicus***				
LSTM4piRNA	**88.50**	88.88	**88.22**	**88.55**	**1.12**
piRNAPredictor	74.91	83.15	71.39	76.82	1.99
GAWE	87.07	**89.85**	85.13	87.42	3.98
piRNN	87.27	88.43	86.43	87.42	2.52
	***M. musculus***				
LSTM4piRNA	**83.34**	**84.07**	**82.86**	**83.46**	**1.28**
piRNAPredictor	73.19	78.02	71.15	74.42	2.23
GAWE	80.00	80.50	79.70	80.10	4.12
piRNN	81.51	80.44	82.20	81.31	2.70
	***C. elegans***				
LSTM4piRNA	**89.25**	**93.80**	**85.98**	**89.72**	**1.12**
piRNAPredictor	78.10	79.05	77.58	78.31	1.66
GAWE	84.30	88.47	81.65	84.93	3.18
piRNN	87.69	91.42	85.07	88.13	2.11

**Table 2:** Performance evaluation on the piRBase v3.0 dataset. Note that we highlight the best performer using boldface font.

Method	*H. sapiens*				
	ACC	SEN	PPV	F1-Score (%)	Log${}_{10}$ (Time)
LSTM4piRNA	**83.81**	**82.81**	**84.49**	**83.64**	**1.87**
piRNAPredictor	70.59	73.09	69.61	71.31	3.22
	***R. norvegicus***				
LSTM4piRNA	**85.25**	**85.57**	**85.03**	**85.30**	**1.55**
piRNAPredictor	72.53	70.30	73.58	71.90	3.18
	***M. musculus***				
LSTM4piRNA	**83.32**	**82.90**	**83.61**	**83.25**	**1.79**
piRNAPredictor	71.77	69.05	73.02	70.98	3.21
	***C. elegans***				
LSTM4piRNA	**88.81**	**92.32**	**86.27**	**89.19**	**1.10**
piRNAPredictor	78.25	79.39	77.62	78.50	1.62
GAWE	82.20	85.03	80.47	82.69	3.20
piRNN	87.45	92.11	84.26	88.01	2.14

Note that only LSTM4piRNA and piRNAPredictor are capable of analyzing datasets for *H. sapiens*, *R. norvegicus*, and *M. musculus* species.

**Table 3:** Statistical summary of the benchmark sequences for each species.

piRBase v1.0	Data Size	Average Length
*H. sapiens*	32,252	28.8
*R. norvegicus*	62,130	28.1
*M. musculus*	100,000	26.9
*C. elegans*	28,219	21.0
**piRBase v3.0**	**Data Size**	**Average Length**
*H. sapiens*	1,000,000	28.5
*R. norvegicus*	1,000,000	28.0
*M. musculus*	1,000,000	27.2
*C. elegans*	30,036	21.0

**Table 4:** Code words for one-hot encoding.

Nucleotide Base	One-Hot Vector
A	[1,0,0,0]
U	[0,1,0,0]
C	[0,0,1,0]
G	[0,0,0,1]
N	[0,0,0,0]

The character “N” represents an uncertain base.

## Data Availability

The web application and source program for LSTM4piRNA are freely accessible at https://lstm4pirna.ee.ncyu.edu.tw (accessed on 19 September 2023).

## Abbreviations

The following abbreviations are used in this manuscript:

LSTM	Long Short-Term Memory
piRNA	Piwi-interacting RNAs
PPV	Positive Predictive Value
SEN	Sensitivity
ACC	Accuracy
